# The role of group IIA secretory phospholipase A2 (sPLA2-IIA) as a biomarker for the diagnosis of sepsis and bacterial infection in adults—A systematic review

**DOI:** 10.1371/journal.pone.0180554

**Published:** 2017-07-03

**Authors:** Toh Leong Tan, Yew Yip Goh

**Affiliations:** Department of Emergency Medicine, Faculty of Medicine, Universiti Kebangsaan Malaysia, Kuala Lumpur, Malaysia; Rutgers University, UNITED STATES

## Abstract

**Introduction:**

This paper investigates the role of Group II Secretory Phospholipase A2 (sPLA2-IIA) as a biomarker for the diagnosis of sepsis and bacterial infection in adults. Sepsis and bacterial infection are common problems encountered by patients in the hospital and often carry adverse outcomes if not managed early.

**Methods:**

Two independent reviewers conducted a comprehensive search using Ovid MEDLINE published from years 1993 to 2016 and SCOPUS published from year 1985 to 2017 to screen for relevant studies. The main inclusion criteria included adult subjects, patients with suspected or confirmed signs of infection and relevant outcomes which looked into the role of sPLA2-IIA in detecting the presence of sepsis and bacterial infection in the subjects.

**Results and discussion:**

Four studies met the inclusion criteria. SPLA2-IIA was found to be effective in detecting the presence of sepsis and bacterial infection in adults. The levels of serum sPLA2-IIA also correlated well with the presence of sepsis and bacterial infection.

**Conclusion:**

This systematic review highlights the role of sPLA2-IIA as a reliable tool to diagnose sepsis and bacterial infection in adult patients. Nonetheless, further studies should be done in the future to provide more compelling evidence on its application in the clinical setting.

## Introduction

Sepsis and bacterial infection are common problems encountered by patients in the hospital. Sepsis carries a poor outcome in terms of mortality and long-term morbidity. Severe sepsis is accountable for one-fifth of all admissions to intensive care units (ICUs) in the United States and contributes to the greatest number of non-cardiac-ICU related deaths[1, 2], despite advances in resuscitation therapy and the use of modern antibiotics [3]. Survivals of sepsis suffer from various complications that arise from organ dysfunction, which leads to severe impairment in their quality of life. The key to sepsis management relies on prompt and accurate diagnosis, as every hour of delay of the appropriate antibiotics has been found to increase mortality by 5 to 10%[3]. Blood culture as the current gold standard in detecting sepsis is a laborious and time-consuming effort which may take up to 48 hours before results are made available. The invariable chances of contaminated specimens and incomplete samples further undermine its feasibility as a rapid and convenient tool in the detection and early recognition of sepsis. Apart from that, only 30–60% of blood cultures will be positive in sepsis and this has led to great levels of diagnostic uncertainty among physicians in the early phases of intervention [4–6]. Thus, it is unsurprising that the constant effort in discovering the most reliable sepsis biomarker has become the focus of intensive research over the past decade. Nonetheless, as patients may develop systemic inflammatory response syndrome (SIRS) for various non-septic reasons, the effort of identifying sepsis accurately from this vast pool of patients remains a challenging task. It is this dilemma that has essentially driven the extensive use of empirical antibiotics, giving rise to the emergence of antimicrobial resistance which is fast becoming a major concern worldwide.

Many studies have suggested that phospholipase A2 (PLA2) plays an essential part in the inflammatory pathway[7]. PLA2 can be classified under a group of acute phase proteins which trigger the host inflammatory response to infection through the liberation of pro-inflammatory arachidonic acid metabolites[7]. As a mediator of inflammation, intracellular PLA2 catalyzes the release of arachidonic acid from membrane phospholipids, thus initiating the synthesis of prostaglandins and leukotrienes[7]. Consequently, this gives rise to several physiological responses such as vasodilation, inhibition of platelet aggregation and chemotaxis. Till date, two distinct groups of extracellular phospholipase A2 have been identified in human plasma, namely group I (pancreatic type), and group II (synovial type)[8]. As a subfamily of enzymes of PLA2, the secretory PLA2 (sPLA2) is present in ten active isoforms. One of the isoforms, i.e. Group II Secretory Phospholipase A2 (sPLA2-IIA), has been found to demonstrate significant association with the presence of sepsis [9–14]. It has also been noted to increase significantly in conditions such as rheumatoid arthritis [15–18], inflammatory bowel diseases (IBD) [19, 20], acute coronary syndrome [21], and asthma [22] which are largely defined by the actions of systemic inflammation. The release of sPLA2-IIA in these conditions is induced by inflammatory cytokines such as interleukin IL-6, IL-1β and tumor necrosis factor (TNF-α), which are key factors in the process of neutrophil adhesion and migration [23–25]. Whilst the exact role of sPLA2-IIA in sepsis is still debatable, several small clinical trials have already shown that plasma levels of sPLA2-IIA exhibited positive correlation with subsequent diagnosis of bacteremia [26, 27]. Hence, in this systematic review, we aim to investigate the role of sPLA2-IIA as a biomarker for the detection of sepsis and bacterial infection in adults. We will also look into the association of sPLA2-IIA with other conventional biomarkers as well as its ability as a risk-stratifying tool for sepsis and bacterial infection.

## Methods

### Eligibility criteria, information sources and electronic search protocol

This systematic review was conducted from December 2016 to March 2017 after obtaining approval from UKM Research Ethical Committee (FF-2007-049). The primary objective was to identify relevant studies regarding the role of sPLA2-IIA as a biomarker for the diagnosis of sepsis and bacterial infection in adults. We adhered to the PRISMA guidelines throughout the development of this systematic review. Medline via Ovid Medline (published between 1993 and 2016) and Scopus (published between 1981 and 2017) were the 2 primary databases used to locate all published literature pertinent to our research topic.

We used the following combination of key words to facilitate our search in MEDLINE: (‘Phospholipase A2, Group IIA’ OR ‘Group II Secretory Phospholipase A2’ OR ‘*PLA2-II*’) AND (‘Sepsis’ OR ‘Bacteria* infect*’) limited by patient's age:—all adult: 19+ years.

As for the search in SCOPUS, the following combination of keywords was used: (‘Phospholipase A2, Group IIA’ OR ‘Group II Secretory Phospholipase A2’ OR ‘*PLA2-II*’) AND (‘Sepsis’ OR ‘Bacteria* infect*’) AND (Adult) AND Limit to “Human”. The exact search term as below:

(((ALL (phospholipase AND a2,group AND iia)) OR (ALL (group AND ii AND secretory AND phospholipase AND a2)) OR (ALL ('*pla2-ii*'))) AND ((ALL (sepsis)) OR (ALL (bacteria* AND infect*)))) AND (adult) AND (LIMIT-TO (EXACTKEYWORD, "Human"))

### Selection of research articles

Studies were eligible for full text review if they fulfill the following set of criteria: 1) the study involved only adult subjects; 2) the patients presented with suspected or confirmed signs of infection; and 3) the primary outcome of the study focused on the role of sPLA2-IIA in detecting the presence of sepsis or bacterial infection in the subjects. Papers related to 1) animal studies; 2) review articles; or 3) articles without complete text were excluded.

### Data extraction and study quality assessment

The process of literature selection was done sequentially in 3 phrases. First, the databases were searched using pre-determined keywords to identify further suitable combination of principle terms. Subsequently, papers with titles and abstract which did not match the inclusion criteria were excluded. In the third phase, the remaining papers were carefully analyzed and any paper that failed to fulfill the requirements was eliminated.

Two reviewers independently read and reviewed the remaining articles after initial screening of titles and abstract. Differences in opinions were resolved through discussion. We applied the QUADAS-2 (Quality Assessment of Diagnostic Accuracy Studies) criteria to assess the all selected articles[28]. Next, the following data was extracted systematically using a data collection form based on: 1) the type of study conducted; 2) a brief description of the sample/population of the study; 3) a brief description of the methodology used; 4) a brief description of the results of the study; 5) the authors’ comments and remarks of the study 6) the risk of bias. A meta-analysis was not performed due to the following reasons: 1) The outcomes, designs, methods, settings and demographics involved in all the 4 studies were too clinically diverse and heterogeneous for quantitative analysis 2) Small number of studies will contribute to wide confidence intervals.

## Results

### Search results

A total of 590 potentially relevant articles were identified, 11 via Medline (S1 Table) and 581 via Scopus. The articles were screened based on their titles and abstract. 11 papers were retrieved from both Medline and Scopus for full text assessment. Seven of these articles were subsequently excluded as they did not meet the primary outcome of the inclusion criteria (S2 Table).

**Fig 1:**
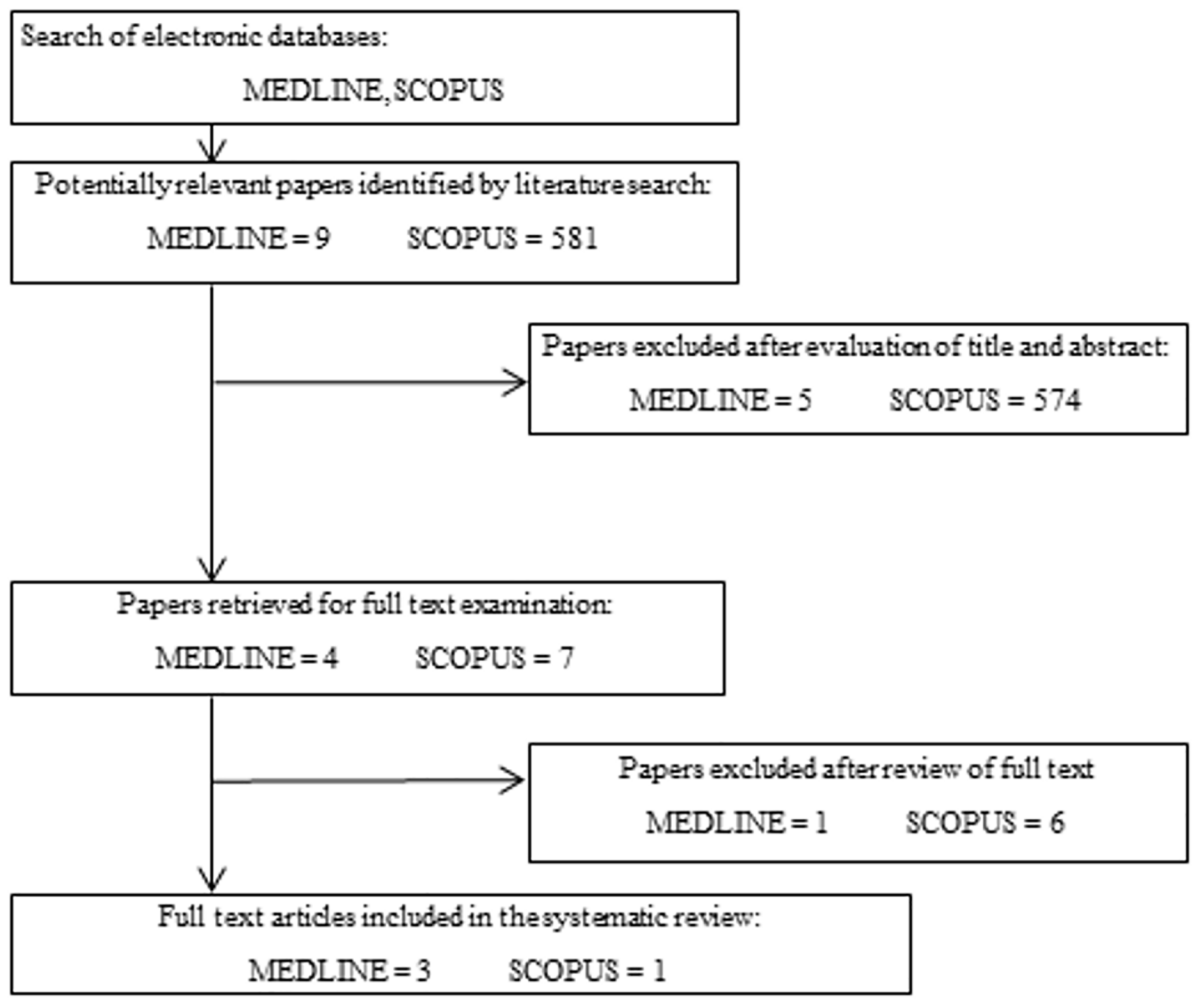
Flow chart shows the process of article selection in this review.

The reviewers came to consensus on the selection of full texts through discussion. After the final review, only 4 articles (three from MEDLINE and one from SCOPUS) were included in this systematic review. The selection process is demonstrated in the flow chart below (Fig 1).

### Study characteristics

The summary of the characteristics of the selected studies is shown in Table 1. All the studies conducted were prospective cross-sectional studies involving only human subjects. Only one study was published before the year 2000[26], whereas, the other three studies were published between the year 2001 to 2016[27, 29, 30]. All the four studies had a sample size of less than 100, ranging from 29 to 80 participants. All the studies involved only adults from both genders. Three out of the four studies had participants with a mean age of 49 to 53[26, 27, 30], while one particular study involved subjects with a mean age of 83[29]. Three of the four studies were carried out in Europe[26, 27, 29] while the remaining article was conducted in Asia[30]. All the four studies were completed within one year (4 months– 10 months), apart from the study conducted by Rintala et al. in 2001 whereby its duration was not mentioned. All the involved subjects in the four studies were recruited from the Department of Infectious Diseases & Medicine[26, 27], medical ward[29] or the Emergency Department of various hospitals[30]. Serial blood samples were taken in two of the four studies[26, 27] while blood samples were taken upon admission in the remaining two studies[29, 30]. The serum concentration of sPLA2-IIA was measured using the ELISA method in all studies.

**Table 1:** The summary of characteristics of the selected studies.

Study	Type of study	Subjects	Methodology	Results	Comments or outcomes
Rintala et al. (1993)	Prospective cross-sectional study	46 (29 Male and 17 Female. Age: 50 ± 23. Country: Finland. Duration: 6 months	**Inclusion criteria:** Axillary temperature minimum of 38°C for more than 12 hours; the presence of a clinically or microbiologically documented infection. **Exclusion criteria:** History of corticosteroid, cytotoxic, or antimicrobial, treatment during the past 1 week; acute pancreatitis, myocardial infarction or trauma in the past 14 days. **Time of sample collection:** Upon admission and at 12, 24 and 48 hours thereafter. **Outcome:** Patients with sepsis, non-septic bacterial infections and viral infections.	**Bacterial infection:** There was no statistically significant difference between the level of sPLA2-IIA in patients with sepsis and blood culture-negative bacterial infections. The levels of sPLA2-IIA in patients with sepsis or blood culture-negative bacterial infections was significantly different from viral infections. (P = 0.0042). Level of sPLA2-IIA was higher in patients with microbiologically over clinically documented infection.	Serum sPLA2-IIA is able to detect the presence of bacterial infection. However, it is unable to determine the severity of bacterial infection. The risk of selection bias is present.
Rintala et al. (2001)	Prospective, cross sectional study	29 (15 Male, 14 Female). Age: 49 ± 22 years. Country: Finland	**Inclusion criteria:** Axillary temperature minimum of 38°C for more than 12 hours, presence of a viral or microbiologically documented bacterial infection **Time of sample collection:** Upon admission and at 24 and 48 hours thereafter. **Outcome:** The presence of bacteremia, non-bacteremia infection or viral infection.	**Bacterial infection:** The overall levels of sPLA2-IIA were higher in patients with infection. The overall levels of sPLA2-IIA in patients with bacteremia were higher than patients with BNBI (p<0.001).	Serum sPLA2-II is able to detect the presence of bacterial infection. The risk of selection bias is present.
Mearelli et al. (2013)	Prospective cross-sectional study	80 (34 Male, 46 Female). Age: Median 83 years (IQR 70–90). Country: Italy. Duration: 4 months.	**Inclusion criteria:** Heart rate > 90 beats per min, fever of more than 38°C or less than 36°C, respiratory rate more than 20 breaths per minute or arterial carbon dioxide tension (PaC02) <32mmHg, white blood cell count more than 12000 cells/mm^3^ or less than 4000 cells/mm^3^. **Exclusion criteria:** Pregnancy, blood drawn for biomarkers after 4 hours from medical ward admission, refusal to provide written informed consent and any antibiotic treatment within the previous 4 weeks. **Time of sample collection:** 4 hours from admission. **Outcome:** Sepsis vs niSIRS (non-infective SIRS), clinical vs microbiological sepsis, blood-culture positive sepsis vs SIRS.	**Sepsis:** Patients with sPLA2-IIA plasma values higher than 6 ng/mL had a 43.5 (95% CI 8–250) odds ratio of having sepsis, compared to those with lower levels. On admission, sPLA2-II level was significantly higher in patients with sepsis than in non-infective SIRS patients. **Bacterial infection:** Serum sPLA2-IIA was significantly higher in blood culture-positive sepsis, compared with SIRS patients (P = 0.003 and 0.013, respectively). There was no difference in biomarker levels between the sepsis subgroups (clinical vs microbiological sepsis) as well as among the type of microorganisms or the sites of infection.	Serum sPLA2-IIA is significantly higher in patients with sepsis. Serum sPLA2-IIA is able to detect the presence of sepsis and bacterial infection. The risk of selection bias is present.
Tan et al. (2016)	Prospective cross-sectional study	51 (26 Male, 25 Female). Age: 53 ± 21. Country: Malaysia. Duration: 10 months.	**Inclusion criteria:** All patients who had a minimum of two SIRS criteria, systolic blood pressure (SBP) less than 90mmHg after a minimum of 30ml/kg crystalloid fluid bolus with suspected bacterial infection. **Exclusion criteria:** Patients with oncological diseases, patients who passed away during the period of recruitment, patients who have been partially treated with antibiotics for at least 4 days, and patients who were transferred to other hospitals. **Time of sample collection:** Upon admission. **Outcome:** Bacterial infection and sepsis	**Sepsis:** Serum sPLA2-IIA has strong correlation with early sepsis in adults. **Bacterial Infection:** Serum sPLA2-IIA is positively correlated in diagnosing bacterial infection.	Serum sPLA2-IIA is able to detect the presence of sepsis and bacterial infection. The risk of selection bias is present.

### Findings on the role of sPLA2-IIA in detecting the presence of sepsis and bacterial infection

Referring to Table 1, all the studies had either used sPLA2-IIA as the sole biomarker or in combination with other biomarkers to examine the relationship between the tested parameters. The SIRS criteria was applied in all four studies as the diagnostic requirements of sepsis whereby 2 or more of the following variables: fever of >38°C (100.4°F) or <36°C (96.8°F), heart rate of >90 beats per minute, respiratory rate of >20 breaths per minute or arterial carbon dioxide tension (PaCO2) of <32mmHg, abnormal white blood cell count (>12000/μL or <4000/μL or >10% immature band forms) must be fulfilled for the presence of sepsis [31, 32].

In 1993, Rintala et al. conducted a prospective study over a span of 6 months in 46 patients who were admitted to the hospital with signs of sepsis, bacterial infection or viral infection. The authors concluded that the serum concentration of sPLA2-IIA was markedly elevated in patients with infection albeit by different degrees over the 3 the classes of infection. The concentration of sPLA2-IIA was found to be highest in patients with sepsis (median, 284.5 μg/L; range, 12.95–1,574 μg/L), followed by blood culture-negative bacterial infections (median, 210.6 μg/L; range, 5.07–1,740 μg/L) and the lowest in those with viral infections (median, 46.78 μg/L; range, 11.46–275.9 μg/L) [26]. Nonetheless, the findings are consistent with the objective of this review that sPLA2-IIA has a significant role in determining the presence of sepsis or bacterial infection in adult patients.

Rintala et al. repeated the study in 2001 which involved 29 patients with the similar set of inclusion criterion to assess the levels of sPLA2-IIA in relation to the presence of bacteremia, blood culture-negative bacterial infection (BNBI) or viral infection. The study concluded that the overall levels of sPLA2-IIA were higher in patients with bacteremia, i.e. blood-culture-positive sepsis as well as in the BNBI group. Higher levels of sPLA2-IIA have also been found in patients with septic shock than those without it. [27]. In 2013, Mearelli et al. conducted a prospective cross-sectional study in 80 eligible patients who fulfilled at least 2 out of 4 variables of the SIRS criteria. In the final 60 patients with either microbiological (n = 20) or clinical sepsis (n = 40), the levels of sPLA2-IIA were significantly higher in septic than non-infected patients with an area under the ROC curve of 0.851 (95% CI 0.742–0.960, P≤0.001). [29]. Patients with sPLA2-IIA plasma values of higher than 6ng/mL were also found to have a 43.5 (95% CI 8–250) odds ratio of having sepsis, compared to those with lower levels. [29] Nevertheless, the authors suggested the need of blinding investigators from biomarker results and posing a validation step of diagnosis to reduce investigator-related bias which could arise from the clinical judgment of sepsis due to the lack of gold standard to which the presumptive diagnosis can be compared.

In 2016, a study was conducted by Tan et al. to evaluate the roles of sPLA2-IIA and CD64 in the diagnosis of sepsis, and to distinguish bacterial from non-bacterial infections. A total of 51 patients were included in the study, with a mean age of 53 ± 21 years. The authors concluded that sPLA2-IIA levels exhibited a good correlation with early sepsis in adults (median 14.5 ± 12.8μg/l, p = 0.001, Mann-Whitney U test). From the study, it was also found that sPLA2-IIA was able to precisely detect sepsis in adults (ROC, AUC = 0.93, 95% CI = 0.83–0.97, Accuracy = 0.88, Kappa = 0.63). These findings were in concordance with Rintala et al. 1993 that the elevation of sPLA2-IIA corresponded with the presence of sepsis in adult patients.

There was a certain degree of selection bias in the studies reviewed. The study population was drawn from participants who were not wholly representative of the target population. The risk for selection bias was significant due to the nature of the subject sampling. All 4 studies used convenient sampling. Prevalence bias was evident as the study population was drawn from participants who were part of a specific subgroup of the disorder of sepsis, e.g. severe sepsis.

## Discussion

This review was systematically done to examine the role of sPLA2-IIA as a biomarker in detecting sepsis and bacterial infection in adults. All 4 studies in this review demonstrated positive correlation between the level of sPLA2-IIA and the presence of sepsis and bacterial infection.

### Bacterial infection

This is consistent with the idea that the serum concentration of sPLA2-IIA is closely related to the inflammatory host response and the activity of specific defense mechanisms that arise in the human body during bacterial invasion. Bacterial infection triggers several processes of acute inflammation which include vascular changes, formation of cellular exudates as well as the release of chemical mediators. In one of the earliest responses, the phospholipid group on the bacteria and host cell membrane lipids is hydrolyzed by the enzyme PLA2, which constitutes a member of acute phase proteins [33]. In 1991, Crowl et al. concluded that human hepatoma cells (HepG2) release sPLA2-IIA in response to various chemotactic factors which include tumor necrosis factor, interleukin-1, and interleukin-6 during an episode of inflammation, therefore implying that sPLA2-IIA could exemplify an acute-phase reactant [34]. The level of sPLA2-IIA is also closely related to phagocytosis and the modification of neutrophil functions, i.e., the release of superoxide, chemotaxis and lysosomal enzymes [7, 35, 36]. Hence, circulatory sPLA2-IIA levels reflect these defense activities in the blood serum and this explains its elevation in the presence of bacterial infection. However, this was contradicted by a few in vitro studies which did not demonstrate any properties of sPLA2-IIA in triggering inflammatory pathways during bacterial infection in mammalian cells as opposed to cytosolic PLA2 (cPLA2) and independent PLA2 (iPLA2)[37–40]. Cytosolic PLA2, an 85-kDa protein, depicts a preference for arachidonic acid and requires micromolar Ca^2+^ concentrations for activity [41] whereas iPLA2 is a Ca^2+^-independent PLA2 subtype of PLA2 isolated from myocardium [42], CHO cells, and macrophages. SPLA2-IIA, on the other hand, are secretory cysteine-rich proteins that function under millimolar concentrations of Ca^2+^ without an inclination towards a specific fatty acid in the sn-2 position of the phospholipid substrate [43]. Nonetheless, the exact mechanism of these subclasses of PLA2 remains the focus of future studies. Apart from that, the time-related kinetics of sPLA2-IIA also confers great clinical significance for the detection of sepsis. According to Rintala et al. 2001, at less than 24 hours, the optimal sensitivity and specificity (both over 80%) of serum sPLA2-IIA levels were achieved at a level of 146 μg/L[27]. This is vital as it is clinically sensible to favor high sensitivity over specificity of a tested parameter in the early diagnosis of bacterial infection. A highly sensitive test is invaluable in assisting physicians with their decision making in the initial stages of admission as whether the administration of antimicrobials would be deemed necessary.

### Sepsis

The reliability of sPLA2-IIA as an early marker of sepsis carries substantial clinical significance. Rapid assessment and diagnosis of sepsis is crucial to prevent adverse outcomes such as severe sepsis, septic shock, acute respiratory distress syndrome (ARDS) and multi-organ dysfunction syndrome (MODS). It can also instigate early commencement of antimicrobials which forms the cornerstone of the management of bacterial-related sepsis as well as non-septic bacterial infections. Besides, the role of sPLA2-IIA in determining the types of infection could be useful to facilitate proper management of the disease condition. According to Rintala et al. 1993, serum levels of sPLA2-IIA in patients with sepsis and blood culture-negative bacterial infections were found to be significantly higher than those with viral infections. As such, prompt introduction of antibiotics should only be considered in the relevant patient groups and this precludes unnecessary use of antibiotics which gives rise to antimicrobial resistance. Apart from that, the degree of elevation of serum sPLA2-IIA was found to correlate with the severity of sepsis and may prove to be useful in predicting its outcomes [44, 45]. Patients who developed septic shock had been shown to depict higher levels of sPLA2-IIA than those who did not [46]. Persistently elevated levels of sPLA2-IIA were also associated with adverse outcomes in sepsis [47]. This could be deduced that the values of sPLA2-IIA were reflective of the various degrees of inflammatory response that occurred in different stages of sepsis. With proper cut-off values available, this may provide significant clinical value for physicians to proceed with the most appropriate management as soon as possible in relation to the severity of the patient’s condition. This could greatly improve the prognosis of sepsis in terms of mortality, morbidity, cost and length of hospital stay.

### Rationale of clinical implementation

To achieve greater practicality in clinical practice, it may be necessary for serum sPLA2-IIA to be combined with other conventional markers of sepsis such as PCT (Procalcitonin), C-reactive protein (CRP), or Erythrocyte sedimentation rate (ESR). The host immune response to sepsis and bacterial infection involves a series of complex inflammatory pathways and it is unlikely that any individual biomarker could be used to precisely classify and predict the outcomes of these conditions. Thus, the idea of a combination of biomarkers may have the theoretical advantage of improving both sensitivity and specificity of the tested outcomes, leading to greater diagnostic accuracy[48]. These are well supported by several findings in the articles included in this review. Rintala et al. 1993 reported that a strong positive correlation was found between the concentrations of sPLA2-IIA and CRP. The levels of sPLA2-IIA also showed significant correlation with the levels of PCT (r = 0.619, p<0.001) and CRP (r = 0.743, p<0.001). Nonetheless, Mearelli et al. 2013 concluded that the combination of sPLA2-IIA with other biomarkers, in Model A, did not improve the accuracy for discrimination of sepsis among SIRS patients, despite reaching a moderate accuracy (AUC = 0.851) when being tested as an individual biomarker. Thus, it is evident that a multi-biomarker panel could presumably offer better diagnostic value over lone biomarkers, but further studies are warranted to provide better understanding of their roles.

### Limitations

This systematic review contains several limitations. Due to the paucity of relevant studies available on the databases, the final selection of this review yielded a seemingly small sample of 4 articles after going through several phases of screening and identification. We understand that the estimation of the magnitude of positive relationships provided by such a small sample size will likely be exaggerated. However, this drawback has been mitigated to its lowest level possible through strict adherence to the inclusion criteria and thorough screening of the articles before elimination. Besides, it should also be noted that all the studies involved in this review possessed a relatively small sample size (<100) to yield ample statistical power. However, the significant results produced by these studies were sufficient to offset this apparent weakness to suggest a fair representation of the interest of the overall population. Apart from that, sPLA2-IIA was not the single primary biomarker included in most of the studies involved and hence did not seem to carry the substantial weightage that we would have preferred. Nonetheless, the results had been thoroughly screened to avoid misrepresentation of the results of other biomarkers. Besides, not all the studies had randomized sampling of subjects as their mode of enrolment. Despite its weaknesses, we acknowledge that the application of consecutive sampling is reasonable when a stringent set of inclusion criteria has been imposed to reduce the number of subject to a degree that it prevents over-generalization of its findings. As such, this will not severely undermine the quality of the selection as it is still typically stronger than other nonprobability methods in controlling sample bias.

## Conclusion

This systematic review highlights the role of sPLA2-IIA as a biomarker for the detection of sepsis and bacterial infection in adults. We conclude that sPLA2-IIA is capable of diagnosing both the aforementioned conditions in adult patients. When used in tandem with other conventional biomarkers such as Lactate[49, 50], CRP[51] and PCT[52, 53], a synergistic effect could be achieved in terms of greater diagnostic sensitivity and specificity as portrayed in the reviewed studies. Besides, the values of serum sPLA2-IIA were found to correlate with the types of infection as well as the severity of sepsis. This substantiates its role as an effective risk-stratifying tool to predict sepsis outcomes in patients. Thus, we suggest that sPLA2-IIA could be potentially applied in the clinical setting to aid physicians in their decision making for early administration of antimicrobial therapy. Nevertheless, we suggest that more studies should be reviewed to yield greater statistical value of the role of sPLA2-IIA in the future.

## Supporting information

S1 Table Medline full electronic search strategy. (DOCX)

S2 Table List of full-text excluded articles. (DOCX)

S3 Table PRISMA checklist. (DOCX)
