# Supplementary material for: The role of group IIA secretory phospholipase A2 (sPLA2-IIA) as a biomarker for the diagnosis of sepsis and bacterial infection in adults—A systematic review
Source: PLoS One. 2017 Jul 3;12(7):e0180554. doi: 10.1371/journal.pone.0180554 (PMC5495423; doi:10.1371/journal.pone.0180554)
Supplement: S1 Table — (DOCX) [file pone.0180554.s001.docx]

**S1 Table. MEDLINE full electronic search strategy**

| [Search ID#](javascript:__doPostBack('ctl00$ctl00$FindField$FindField$historyControl$ReorderHistoryLink','')) | **Search Terms** | **Search Options** | **Actions** |
| --- | --- | --- | --- |
| S8 | (S1 OR S2 OR S3) AND (S4 OR S5) | **Narrow by SubjectAge:**- all adult: 19+ years  **Search modes** - Boolean/Phrase | [**View Results**](javascript:__doPostBack('ctl00$ctl00$FindField$FindField$historyControl$HistoryRepeater$ctl00$linkResults','')) (9)  [**View Details**](javascript:showShDetails(%22ctl00_ctl00_FindField_FindField_historyControl_ctrlPopup%22,%20%22S8%22);)  [**Edit**](http://web.a.ebscohost.com/Legacy/Views/UserControls/Ehost/) |
| S7 | (S1 OR S2 OR S3) AND (S4 OR S5) | **Search modes** - Boolean/Phrase | [**View Results**](javascript:__doPostBack('ctl00$ctl00$FindField$FindField$historyControl$HistoryRepeater$ctl01$linkResults','')) (16)  [**View Details**](javascript:showShDetails(%22ctl00_ctl00_FindField_FindField_historyControl_ctrlPopup%22,%20%22S7%22);)  [**Edit**](http://web.a.ebscohost.com/Legacy/Views/UserControls/Ehost/) |
| S6 | S1 OR S2 OR S3 | **Search modes** - Boolean/Phrase | [**View Results**](javascript:__doPostBack('ctl00$ctl00$FindField$FindField$historyControl$HistoryRepeater$ctl02$linkResults','')) (193)  [**View Details**](javascript:showShDetails(%22ctl00_ctl00_FindField_FindField_historyControl_ctrlPopup%22,%20%22S6%22);)  [**Edit**](http://web.a.ebscohost.com/Legacy/Views/UserControls/Ehost/) |
| S5 | Bacteria* infect* | **Search modes** - Boolean/Phrase | [**View Results**](javascript:__doPostBack('ctl00$ctl00$FindField$FindField$historyControl$HistoryRepeater$ctl03$linkResults','')) (107,746)  [**View Details**](javascript:showShDetails(%22ctl00_ctl00_FindField_FindField_historyControl_ctrlPopup%22,%20%22S5%22);)  [**Edit**](http://web.a.ebscohost.com/Legacy/Views/UserControls/Ehost/) |
| S4 | Sepsis | **Search modes** - Boolean/Phrase | [**View Results**](javascript:__doPostBack('ctl00$ctl00$FindField$FindField$historyControl$HistoryRepeater$ctl04$linkResults','')) (104,981)  [**View Details**](javascript:showShDetails(%22ctl00_ctl00_FindField_FindField_historyControl_ctrlPopup%22,%20%22S4%22);)  [**Edit**](http://web.a.ebscohost.com/Legacy/Views/UserControls/Ehost/) |
| S3 | *PLA2-II* | **Search modes** - Boolean/Phrase | [**View Results**](javascript:__doPostBack('ctl00$ctl00$FindField$FindField$historyControl$HistoryRepeater$ctl05$linkResults','')) (115)  [**View Details**](javascript:showShDetails(%22ctl00_ctl00_FindField_FindField_historyControl_ctrlPopup%22,%20%22S3%22);)  [**Edit**](http://web.a.ebscohost.com/Legacy/Views/UserControls/Ehost/) |
| S2 | Group II Secretory Phospholipase A2 | **Search modes** - Boolean/Phrase | [**View Results**](javascript:__doPostBack('ctl00$ctl00$FindField$FindField$historyControl$HistoryRepeater$ctl06$linkResults','')) (12)  [**View Details**](javascript:showShDetails(%22ctl00_ctl00_FindField_FindField_historyControl_ctrlPopup%22,%20%22S2%22);)  [**Edit**](http://web.a.ebscohost.com/Legacy/Views/UserControls/Ehost/) |
| S1 | Phospholipase A2, Group IIA | **Search modes** - Boolean/Phrase | [**View Results**](javascript:__doPostBack('ctl00$ctl00$FindField$FindField$historyControl$HistoryRepeater$ctl07$linkResults','')) (70)  [**View Details**](javascript:showShDetails(%22ctl00_ctl00_FindField_FindField_historyControl_ctrlPopup%22,%20%22S1%22);)  [**Edi**](http://web.a.ebscohost.com/Legacy/Views/UserControls/Ehost/)t |
