# Supplementary material for: The role of group IIA secretory phospholipase A2 (sPLA2-IIA) as a biomarker for the diagnosis of sepsis and bacterial infection in adults—A systematic review
Source: PLoS One. 2017 Jul 3;12(7):e0180554. doi: 10.1371/journal.pone.0180554 (PMC5495423; doi:10.1371/journal.pone.0180554)
Supplement: S2 Table — (DOCX) [file pone.0180554.s002.docx]

**S2 Table. List of full-text excluded articles**

Description of studies selected using MEDLINE database search strategy.

Descriptors: ‘Phospholipase A2, Group IIA’ OR ‘Group II Secretory Phospholipase A2’ OR ‘*PLA2-II*’ AND ‘Sepsis’ OR ‘Bacteria* infect*’ narrowed by subject age: - all adult: 19+ years.

As for the search in SCOPUS, the following combination of keywords was used: (‘Phospholipase A2, Group IIA’ OR ‘Group II Secretory Phospholipase A2’ OR ‘*PLA2-II*’) AND (‘Sepsis’ OR ‘Bacteria* infect*’) AND (Adult) AND Limit to “Human”. The exact search term as below:

( ( ( ALL ( phospholipase AND a2,group AND iia ) ) OR ( ALL ( group AND ii AND secretory AND phospholipase AND a2 ) ) OR ( ALL ( '*pla2-ii*' ) ) ) AND ( ( ALL ( sepsis ) ) OR ( ALL ( bacteria* AND infect* ) ) ) ) AND ( adult ) AND ( LIMIT-TO ( EXACTKEYWORD , "Human" ) )

| No. | Authors | Title of the articles | Included | Excluded | Reason of exclusion |
| --- | --- | --- | --- | --- | --- |
| 1. | Tan TL et al.*,* 2016 | CD64 and Group II Secretory Phospholipase A2 (sPLA2-IIA) as Biomarkers for Distinguishing Adult Sepsis and Bacterial Infections in the Emergency Department | X |  |  |
| 2. | Mearelli F et al., 2014 | Heterogeneous models for an early discrimination between sepsis and non-infective SIRS in medical ward patients: a pilot study | X |  |  |
| 3. | Di Somma S et al*.,* 2013 | Opinion paper on innovative approach of biomarkers for infectious diseases and sepsis management in the emergency department. |  | X | This is an opinion paper |
| 4. | Dajak M et al*. ,* 2006 | Prognostic value of phospholipase A2 group II, C-reactive protein and Simplified Acute Physiological Score II in intensive care patients. |  | X | The outcome of the study did not meet our primary outcome. |
| 5. | Rintala EM et al., 2000 | Bactericidal/permeability-increasing protein (BPI) in sepsis correlates with the severity of sepsis and the outcome. |  | X | The objectives of this study did not meet the primary objective of our review. |
| 6. | Aittoniemi J et al. , 1997 | Serum mannan-binding lectin (MBL) in patients with infection: clinical and laboratory correlates. |  | X | The objectives of this study did not meet the primary objective of our review. |
| 7. | Rintala EM et al. *,* 1995 | Endotoxin, interleukin-6 and phospholipase-A2 as markers of sepsis in patients with hematological malignancies. |  | X | The inclusion criteria was not met. All the subjects had hematological malignancies. |
| 8. | Rintala EM et al*. ,* 1993 | Group II phospholipase A2 in sera of febrile patients with microbiologically or clinically documented infections. | X |  |  |
| 9. | Rintala EM et al*. ,* 1993 | Synovial-type (group II) phospholipase A2 in serum of febrile patients with haematological malignancy. |  | X | The inclusion criteria was not met. All the subjects had hematological malignancies |
| 10 | Groeneveld et al., 2008 | The role of the innate immune response in hospital- versus community-acquired infection in febrile medical patients |  | X | The inclusion criteria was not met. All the subjects had innate immunity. |
| 11 | Groeneveld et al., 2003 | Circulating inflammatory mediators predict shock and mortality in febrile patients with microbial infection |  | X | The outcome of the study did not meet our primary outcome. |
| 12 | Grönroos JO et al. , 2002 | Bactericidal group IIA phospholipase A2 in serum of patients with bacterial infections. |  | X | The objectives of this study did not meet the primary objective of our review. Antibacterial properties of Group IIA Phospholipase A2 |
| 13 | Rintala et al., 2001 | Early identification of bacteremia by biochemical markers of systemic inflammation | X |  |  |
| 14 | Guidet et al., 1996 | Secretory non-pancreatic phopholipase A2 in severe sepsis: Relation to endotoxin, cytokines and thromboxane B2 |  | X | The objectives of this study did not meet the primary objective of our review. Antibacterial properties of Group IIA Phospholipase A2 |
| 15 | Nakae et al., 1996 | Nitrite/nitrate (NOx) and type II phospholipase A2, leukotriene B4, and platelet-activating factor levels in patients with septic shock |  | X | The objectives of this study did not meet the primary objective of our review. Antibacterial properties of Group IIA Phospholipase A2 |
| Total number of articles | | | 4 | 11 |  |
